# Metabolite coupling in genome-scale metabolic networks

**DOI:** 10.1186/1471-2105-7-111

**Published:** 2006-03-06

**Authors:** Scott A Becker, Nathan D Price, Bernhard Ø Palsson

**Affiliations:** 1 Department of Bioengineering, University of California, San Diego, 9500 Gilman Drive, La Jolla, California, 92093, USA; 2 Institute for Systems Biology, 1441 North 34th Street, Seattle, Washington, 98103, USA

## Abstract

**Background:**

Biochemically detailed stoichiometric matrices have now been reconstructed for various bacteria, yeast, and for the human cardiac mitochondrion based on genomic and proteomic data. These networks have been manually curated based on legacy data and elementally and charge balanced. Comparative analysis of these well curated networks is now possible. Pairs of metabolites often appear together in several network reactions, linking them topologically. This co-occurrence of pairs of metabolites in metabolic reactions is termed herein "metabolite coupling." These metabolite pairs can be directly computed from the stoichiometric matrix, **S**. Metabolite coupling is derived from the matrix **ŜŜ**^T^, whose off-diagonal elements indicate the number of reactions in which any two metabolites participate together, where **Ŝ **is the binary form of **S**.

**Results:**

Metabolite coupling in the studied networks was found to be dominated by a relatively small group of highly interacting pairs of metabolites. As would be expected, metabolites with high individual metabolite connectivity also tended to be those with the highest metabolite coupling, as the most connected metabolites couple more often. For metabolite pairs that are not highly coupled, we show that the number of reactions a pair of metabolites shares across a metabolic network closely approximates a line on a log-log scale. We also show that the preferential coupling of two metabolites with each other is spread across the spectrum of metabolites and is not unique to the most connected metabolites. We provide a measure for determining which metabolite pairs couple more often than would be expected based on their individual connectivity in the network and show that these metabolites often derive their principal biological functions from existing in pairs. Thus, analysis of metabolite coupling provides information beyond that which is found from studying the individual connectivity of individual metabolites.

**Conclusion:**

The coupling of metabolites is an important topological property of metabolic networks. By computing coupling quantitatively for the first time in genome-scale metabolic networks, we provide insight into the basic structure of these networks.

## Background

Cellular metabolism is an extensively studied process that is essential to the survival of any free-living organism. The metabolic process can be characterized as a set of biochemical transformations, each of which involves the consumption of one or more metabolites and the production of one or more metabolites. Subject to the law of mass conservation, the net sum of elements and electrical charge is conserved in each reaction and thus in the network as a whole. Each transformation by definition must involve more than one metabolite. Herein, we define "metabolite coupling" as the appearance of a pair of metabolites in the same biochemical transformation.

Any examination of carefully balanced biochemical transformations immediately displays the ubiquity of the proton (H^+^) and water in metabolic reactions. The ubiquity of the proton in metabolic networks is interesting in light of the fact that at standard pH a typical bacterial cell contains approximately 60 free protons. Assuming pH = 7 [1] and the volume of the cell is equal to 1 μm^3^, the number of free protons in solution is equal to (10^-7 ^mol H^+^/L)(6.022 × 10^23 ^molecules/mol)(cell volume L); unit conversions yield the final answer. Thus, through the medium of water, the protons must be rapidly shuttled around the cell as metabolic transfers are taking place. Although the inclusion of protons is not complete in most metabolic pathway databases, protons have been included where appropriate in the networks analyzed in the present work. While the inclusion of protons in genome scale metabolic reconstructions is not frequent, it does allow for the computation of important effects of H^+ ^balancing on network functions. For example, the change in pH of the growth media for cultures of *Escherichia coli *was predicted by a balanced model [2]. Careful elemental and charge balancing of the human mitochondrial metabolic network also allowed for the computational determination of ATP yield [3].

With the exception of the proton and water, the most commonly coupled metabolites are those generally referred to as cofactors (i.e. ATP/ADP, NAD^+^/NADH). Cofactors fulfil a range of roles in cells, from transferring energy and redox potential to carrying key metabolic intermediates. In some cases, different common cofactors can serve the same purpose – ATP and GTP can both deliver energy; NADH and NADPH can both supply reducing power – but one is often preferred for a given purpose in an organism. For example, NADH is more commonly used during the oxidative reactions that comprise catabolism whereas NADPH is preferred for the reductive reactions that are involved in anabolism [4]. The exact use of cofactors in metabolic reactions is typically not fully defined in genome annotations, and thus metabolic pathways derived from a sequence-based annotation may not correctly specify the cofactor used in a particular reaction in a network [5].

Topological analyses of the cellular metabolism of microorganisms have generated significant interest recently [6]. Metabolic networks have been shown to exhibit scale-free behavior, where the number of reactions in which a given metabolite participates follows a power-law distribution, meaning that the probability of a metabolite having *k *connections to other metabolites is approximately equal to k^-γ^, where γ is constant for a given network [7,8]. The reaction fluxes in the central metabolism of *E. coli *have been shown to behave in a similar manner; the probability that a reaction has a given flux *v *is proportional to (*v*+*v*_0_)^-α^, with *v*_0 _and α constant [9]. The overall hierarchical and modular organization of metabolic networks has been demonstrated [10]. Protein domain networks also have been suggested to have a scale-free nature [11]. The methodology and principal results of the topological analysis of biological networks has been recently reviewed [6]. More recently, it has been asserted that a power law is a natural consequence of a high variability process, much as the central limit theorem dictates a Gaussian distribution for lower variability processes [12].

Metabolic networks of differing levels of detail and accuracy can be encoded using a variety of formalisms, including (hyper)graphs [7], petri nets [13], and stoichiometric matrices [14,15]. The level of detail of a metabolic network is primarily determined by the method of reconstruction, independent of the specific encoding used. For example, networks generated in a semi-automated fashion principally from metabolic pathway databases enable high-throughput reconstructions for many organisms but sacrifice accuracy with regard to fine details such as cofactor preference (NADH vs. NADPH) and proton balancing. In contrast, the metabolic networks considered herein were manually reconstructed from diverse sources, including metabolic databases, legacy biochemical data, and physiological data [16]. They are curated to the point where they can be used for computation, and the subsequent comparison of these computations to experimental data provides further evidence as to the quality of the reconstructions [17,18]. These networks were initially published using the stoichiometric matrix formalism. Furthermore, all of the metabolic networks analyzed herein were scrutinized for cofactor preference on a reaction-by-reaction basis as well as elementally and charge balanced before the networks were initially published, meaning that each reaction was examined to determine if the addition of protons was necessary to enforce the conservation of elements and charge at cellular pH. A study of metabolite coupling requires both this balancing and the most accurate assignment of cofactors to reactions possible for each organism.

In this paper, we present a topological analysis of metabolite coupling in genome-scale metabolic networks. We find that most pairs of metabolites never participate in a reaction together, smaller numbers of pairs occur together in a few reactions, and a select few pairs, such as cofactors, occur in a significant fraction of the reactions in the network, in many cases more often than would be expected based on random connectivity. This work is distinct from the previous analyses because we consider the consequences of *pairs *of metabolites occurring together on a genome scale, rather than individual metabolites. The motivation for studying metabolite pairs is that many biomolecules are of more interest as pairs than as single molecules. For example, ATP is most important because of its ability to couple with ADP, P_i _and H^+ ^in reactions that transfer the phosphate moiety as a form of energy currency for a cell. Studies of metabolite coupling can be used to highlight such chemically-based network properties as they appear throughout the entire network for multiple organisms. Because we focus on coupling, having essential detail in cofactor usage is essential. This necessitates that we use only the hand-curated metabolic networks that are available for a select few microorganisms and the human cardiac mitochondrion.

## Results and discussion

### Basic network properties

We characterized six metabolic networks based on topological features, with representatives from each of the three primary domains of life. The metabolic networks considered represent three bacteria (*E. coli *[2], *Helicobacter pylori *[19], *Staphylococcus aureus *[20]), one member of the archea (*Methanosarcina barkeri *[21]), one unicellular eukaryote (*S. cerevisiae *[22]), and one human cellular organelle (cardiac mitochondrion [3]). These are, to the best of our knowledge, the only genome-scale metabolic networks that are manually curated as well as elementally and charge balanced available to date.

**Figure 1:**
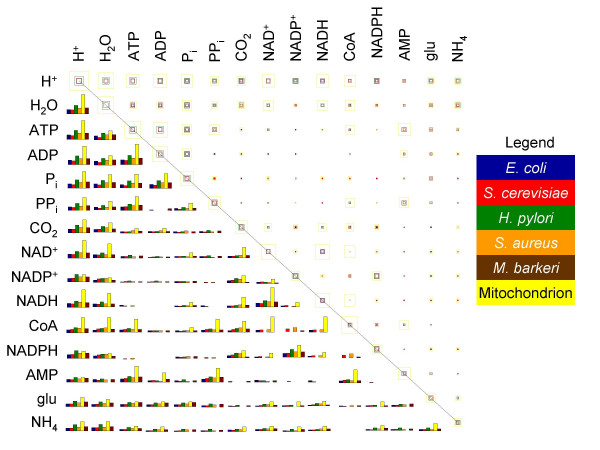
**The coupling of highly connected metabolites**. The first 15 rows and columns of **M **are shown for all six networks analyzed. The box and bar colors represent the network: *E. coli *(blue), *S. cerevisiae *(red), *H. pylori *(green), *S. aureus *(orange), mitochondrion (yellow), *M. barkeri *(brown). The box size and bar height represent the number in each particular element of **M**, scaled by a log transform and normalized by the network size. Larger boxes or taller bars of a given color indicate that the pair of metabolites in question participate in relatively more reactions together. Reading down the rows or across the columns gives the most connected metabolites in order, averaged over all six networks.

We computed the symmetric metabolite coupling matrix **M **for each network by multiplying the binary form of the stoichiometric matrix **S **by its transpose (see Methods for details). Each diagonal element of **M **(m_ii_) represents the number of reactions in which a particular metabolite appears and each off-diagonal element (m_ij_, i ≠ j) represents the number of reactions in which two particular metabolites appear together. Figure 1 shows a graphical representation of the first 15 rows and columns of **M **for all six networks. Both the box size and the histogram height are logarithmically scaled and normalized by the number of unique metabolite pairs that occur in each network. This scaling and normalization allows for the visualization of pairs of metabolites that participate in anywhere from zero to several hundred reactions together in any given network. The color of each box and bar represents one particular network. Any matrix constructed by the multiplication of another matrix with its transpose is symmetric, and thus the points above the diagonal of **M **are identical to those below; thus, each histogram below the diagonal corresponds to one set of colored boxes above the diagonal. It is notable that, of the 15 most connected metabolites, only three pairs never couple in the six networks analyzed here: ADP/NADH, ADP/NADPH, and NH_4_/CoA. The first two metabolite pairs that never couple are perhaps surprising, because it is well known that cells use electron carriers to phosphorylate ADP. However, because this process occurs in a series of reactions instead of a single reaction, these metabolites are not considered to be coupled. The computation of second-degree coupling (where two metabolites are coupled if they couple with at least one common third metabolite) would find this relationship, but would also introduce significant difficulties because many metabolite pairs would be second-degree coupled solely because they are individually coupled with the proton or water.

**Figure 2:**
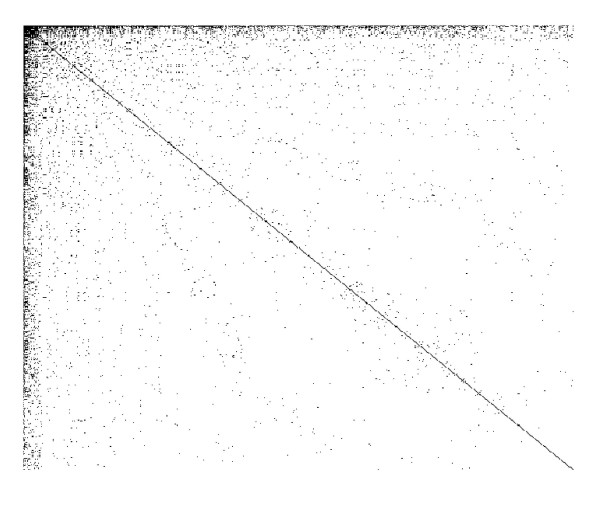
**The binary form of the metabolite coupling matrix for *E. coli***. The binary form of **M **for *E. coli *is shown. Approximately 98% of the values in **M **are zero, so 98% of the image is white. Off-diagonal black points only appear where two metabolites occur in at least one reaction together. The diagonal and the prominence of coupling in the first few rows and columns (most connected metabolites) is clearly illustrated. As in figure 2, each row and column represents a particular metabolite.

Basic properties of each network and its corresponding **M **are shown in table 1. The metabolic network of *E. coli *has 621 compounds and thus its **M **is a square symmetric matrix with 621^2 ^elements. Each compound in the network must participate in at least one reaction, so each diagonal element of **M **must be greater than zero. However, there is no general topological reason that an arbitrary pair of metabolites must participate in any reaction together, so zeroes are feasible for off-diagonal elements. In fact, the density of **M **for E. coli is 2.2%, meaning that approximately 98% of the (((621^2^)/2)-621) unique off-diagonal elements are zero. Figure 2 is a binary representation of **M **for *E. coli*, constructed by keeping all zeros in place (white spaces) and replacing all non-zeros with 1 (black points), that clearly demonstrates this sparsity. Smaller networks tend to have a denser **M**.

### Metabolite coupling

**Figure 3:**
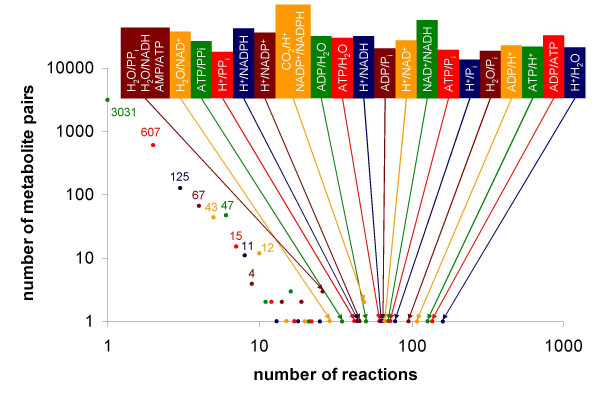
**The distribution of metabolite coupling in *E. coli***. The number of metabolite pairs that occur together in each possible number of reactions is plotted on a log-log scale. There are many metabolite pairs that never occur together, and there are many particular numbers of reactions in which no metabolite pairs occur, but these are not shown because zero does not appear on a log-log plot. The points and most coupled metabolite pairs are colored to improve clarity and readability, but they are all in the *E. coli *network. The top four metabolite pairs, located at the right of the figure, all occur in over 100 reactions together.

**Figure 4:**
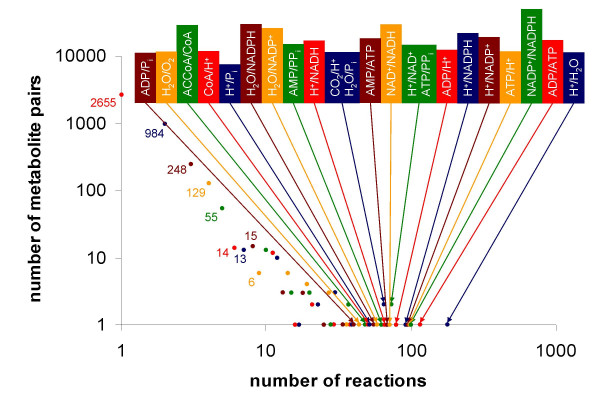
**The distribution of metabolite coupling in *S. cerevisiae***. Identical to figure 3, but using the *S. cerevisiae *network. The general trends are similar but the metabolite identities and precise distribution differ.

For each of the six networks, we determined the number of reactions in which each possible pair of metabolites is coupled. We defined two metabolites as coupled in a given reaction if they both occur in that reaction, in contrast to the recently presented concept of metabolite concentration coupling analysis which studies metabolites whose concentrations are linked by network stoichiometry [23]. The distribution of metabolite coupling is shown for *E. coli *(figure 3) and *S. cerevisiae *(figure 4). Most pairs of metabolites are never coupled and cannot be shown on a log-log plot. Many pairs of metabolites occur together in only one reaction and are illustrated by the leftmost data point of each figure. The pairs of coupled metabolites that occur in many reactions together are the rightmost points in each plot and represent pairs of metabolites that are either small and ubiquitous (e.g. H^+^, PP_i_) or traditionally referred to as cofactors (e.g. NAD^+^, NADH). In the six networks considered, the two most common metabolite pairs are always H^+^/H_2_O and ATP/ADP. The pair ATP/ADP is ranked first in *H. pylori *and *S. aureus *but second in the remaining four networks. The identity and order of coupled metabolite pairs diverges after these two pairs, as illustrated by the plots for the two model microorganisms, *E. coli *and *S. cerevisiae*.

**Figure 5:**
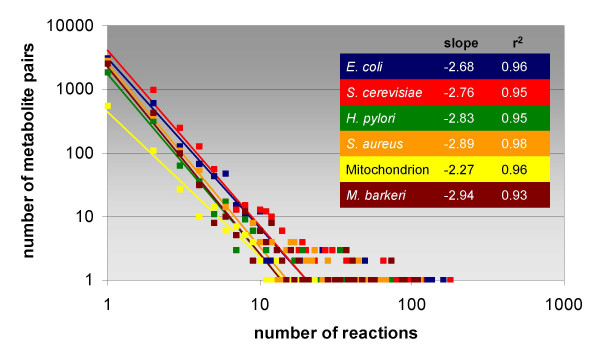
**The distribution of metabolite coupling in all networks**. The data for all six networks is plotted in the same way as in figures 3 and 4, but colored to represent each network. The best-fit lines are constructed as described in the text. The r^2 ^values indicate how good each network fits a line of the slope indicated in the figure.

The number of reactions in which each possible combination of two metabolites participates approximates a line on a log-log scale in all networks studied, with average slopes between -2 and -3 (figure 5). The slope of the best-fit line (linear on a log-log scale) was determined for each network from the left-most 10 points and is indicated in the figure legend, along with the corresponding r^2 ^value. While the dominance of certain cofactor pairs in metabolic networks was known, the strong fit to a line for the less connected pairs demonstrates significant regularity in the use of pairs of metabolites in metabolic networks. The slope of this line provides a quantitative measure to describe the organization of metabolite coupling in the network and can be used to compare coupling properties of different networks of various sizes. Because the coupling relationships fit a line so well, it is possible in principle to determine the relative dominance of certain pairs of metabolites across networks. A smaller (negative) slope generally implies that there are either fewer metabolite pairs that only appear once or twice or that there are more pairs of metabolites that participate in an intermediate number of reactions (for example, 5 to 10). A smaller slope suggests that relatively more metabolite pairs influence more than one reaction and link reactions together.

### Metabolite connectivity

**Figure 6:**
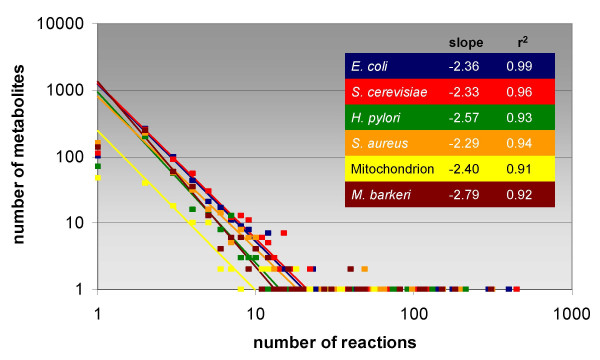
**The distribution of metabolite connectivity in all networks**. Identical to figure 5, but plotting the occurrence of individual metabolites in a given number of reactions instead of metabolite pairs.

For each reconstructed metabolic network, we calculated the number of reactions in which an individual metabolite occurs, m_ii_, as a measure of its individual connectivity in each network (figure 6). The first reconstructed genome-scale network for *Haemophilus influenzae *metabolism [24] suggested that this property had a power-law distribution and fit a line on a log-log scale. Similar calculations made for many networks [7] using a somewhat different network formalism described earlier yielded similar results. The fit to a line in figure 6 begins after the number of occurrences is two or greater, since a metabolite must participate in at least two reactions to be involved in reactions that can be active at steady state. This result is different from the graph-based approaches that plot in-degree and out-degree separately [7], essentially converting all metabolites that participate in two reactions into two occurrences of one reaction each.

Furthermore, the slope of each line for single metabolite connectivities (figure 6) is different than the slope of the corresponding line for metabolite coupling (figure 5). When the ratio of the slope for metabolite coupling to the slope for single metabolite participation is computed, the results range from 0.95 (mitochondrion) to 1.54 (*S.aureus*) with a mean of 1.11 and standard deviation of 0.10. Furthermore, when the slopes are rank ordered from greatest to least, it is observed that the networks are ordered differently when considering single metabolites than when considering metabolite coupling. Thus, the widely differing slope ratios and differential ordering indicates that the distribution of metabolite coupling (off-diagonal elements in **M**) is not simply a direct result of the distribution of metabolite connectivity (diagonal elements in **M**).

### Relation between metabolite coupling and metabolite connectivity

Although the slopes of best-fit lines vary considerably for single metabolites and metabolite pairs, there is an important relationship between metabolite coupling and individual metabolite connectivity because a particular metabolite cannot be highly coupled with other metabolites if it does not appear in many reactions. Thus, each off-diagonal element of **M **(m_ij_) is subject to both of the following criteria:

m_ij _≤ m_ii_

m_ij _≤ m_jj_

**Figure 7:**
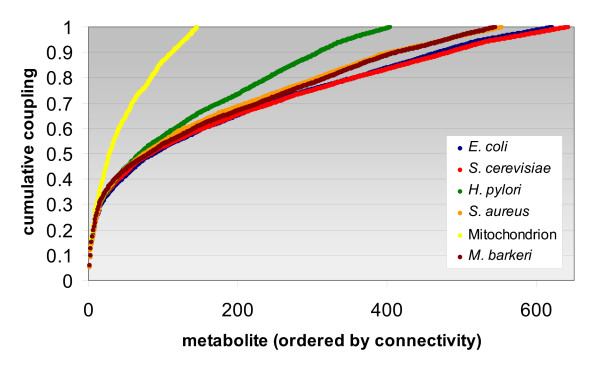
**Coupling interactions vs. metabolite connectivity**. The cumulative number of coupling interactions accounted for by each metabolite, rank ordered by connectivity in each network, is plotted against the number of metabolites.

The metabolite coupling and connectivity is shown graphically for *E. coli *in figure 2, with the rows and columns ordered by overall connectivity. The most connected metabolites couple with the greatest number of other metabolites, as shown by the large number of black points toward both the top and the left side of the figure. The number of black points in each row (not counting the one black point on the diagonal) is the number of unique compounds with which a given compound couples. For example, in E. coli, hydrogen couples with 479 metabolites and thus there are 479 black points in row 1 (not counting the diagonal). The matrix is symmetric, so there are also 479 black points in column 1. Figure 7 further illustrates that the most connected metabolites couple with considerably more different metabolites than less-connected metabolites. The sum of each row of figure 2, added to the sum of all previous rows and normalized gives a data point in figure 7. For all networks, the first point, representing hydrogen, is between 0.05 and 0.06 in figure 7, signifying that hydrogen accounts for between 5% and 6% of all coupling interactions. The second point, representing the percentage of coupling interactions involving either hydrogen or water, is close to 0.10 (10%) in all networks. The number of metabolites that couple with of the top 15 most connected metabolites is listed in table 2, the second column of which is the numerical result of summing across a row in figure 2. It is not surprising that metabolites like the proton and ATP, which each occur in many reactions, couple with many different metabolites. In figure 7, the nearly constant slope in the middle region of the curve (between approximately metabolite 100 and 500) for all networks except the mitochondrion is interesting, however. While it takes fewer than the most-connected 100 metabolites to account for 50% of the coupling, it takes between 300 and 500 metabolites to account for 90% of the coupling in any given network, excluding the mitochondrion. Furthermore, each of the 300 to 500 metabolites in that region between 50% and 90% participates roughly equally in the cumulative coupling interactions; that is, each of these metabolites couples with a roughly equal number of other metabolites. This is likely due to the simple fact that most of these metabolites each participate in two reactions. The slight decrease in slope for the final 50 to 100 metabolites suggests that the least connected metabolites each account for slightly less coupling, which is unsurprising since most of these metabolites occur in only one reaction, and are dead-ends in their respective networks.

The mitochondrion is a special case that does not conform well to the previous generalizations. An examination of the mitochondrial metabolic network shows that, relative to its size, the mitochondrion contains a disproportionately high number of metabolites that are highly coupled in other networks. Whereas the 15 most connected metabolites represent only 2.4% of the 621 metabolites present in *E. coli*, they account for over 10% of the mitochondrion's 145 metabolites. We present the results for the mitochondrion in the interest of completeness, but caution that it may be difficult to extract meaning from a direct comparison to other, more comprehensive, metabolic networks. While normalization of the results to some measure of network size would bring the data in figure 7 into closer agreement, it would not change the simple fact that the mitochondrial network is fundamentally more limited in scope and over-represents highly connected metabolites relative to the genome-scale matrices. This high connectivity and coupling within the mitochondrion suggests that the individual metabolic reactions therein are more interrelated and may affect each other to a greater extent, on average, than those within a genome-scale bacterial metabolic network.

### Preferentially coupled metabolite pairs

**Figure 8:**
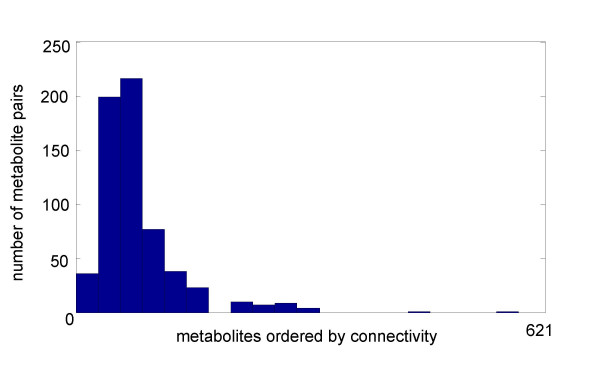
**Preferentially coupled metabolite pairs in *E. coli***. The metabolite pairs in *E. coli *that are greater than 99% likely to be preferentially coupled in a non-random fashion are summed by row/column and binned. The ordering of the rows in this figure is identical to figure 3, proceeding from most to least connected metabolites from left to right. Each preferential coupling is counted twice, once for each metabolite involved. The most connected metabolites occupy the bins on the left, and clearly account for most of the preferential coupling detected.

There are some pairs of metabolites that occur in reactions together at a higher rate than would be expected given their individual connectivities. In order to locate these pairs, we used a Monte Carlo approach to uniformly sample the possible values of each off-diagonal element of the metabolite coupling matrix given that the diagonal (the frequency of a given metabolite's participation in the network) remained unchanged. Based on the two diagonal elements corresponding to each off-diagonal element (m_ii _and m_jj _correspond to m_ij_), each off-diagonal element is assigned a random, feasible value. Complete details are in the methods section. After doing this many times for each pair of metabolites in each network, it is possible to determine which metabolite pairs in each network are coupled more than expected for a random network with the same individual connectivity. The pairs that never couple as often in 10,000 randomizations as they do in the real network are listed for *E. coli *in table 3. The spread of preferentially coupled metabolite pairs (those that couple more often in the real network than in 99% of the randomizations) is graphically represented for *E. coli *in figure 8. The very existence of preferentially coupled metabolites demonstrates the non-random organization of metabolite coupling. This non-random coupling can be a function of fundamental chemical principles or biological needs, such as the general need for both ATP and ADP to transfer a phosphate moiety.

Furthermore, a number of less connected metabolites preferentially couple, demonstrating that this effect is not limited to the highly-connected metabolites. Although many of the preferentially coupled pairs are clustered toward the left side of figure 8 and correspond to the most connected metabolites, the remainder are spread throughout much of the figure, and as a result, the network as a whole. Thus, participating in many reactions is not necessary to detect non-random preferential coupling. This suggests that metabolites with low connectivity overall in the network are still tightly connected to certain other metabolites, often through shared chemical structural properties. Examination of the list presented in table 3 supports this assertion. The proton couples preferentially with several metabolites that gain or lose a proton during the course of balanced biochemical transformations. The adenine and phosphate containing metabolites preferentially couple in a number of cases. Sugars preferentially couple with other sugars, and fatty acids preferentially couple with other fatty acids.

### Preferentially uncoupled metabolite pairs

The same Monte Carlo randomization used to locate overly coupled pairs of metabolites was also used to find metabolites that occur together less often than expected. This procedure located relatively high connectivity metabolites (those that individually participate in many reactions) that do not couple as often as they would in a network with random coupling; results are shown for *E. coli *in table 4. For example, this procedure indicates that ATP and NADH couple less than expected based on the number of reactions in which they participate together in every genome-scale network. This preferential uncoupling between ATP and NADH allows the network more flexibility in controlling energy and redox needs. Although these two metabolites never occur together in any reaction in the mitochondrion, having even 95% confidence that this is a non-random result would require that they participate in fewer than zero reactions together and thus this observation was not statistically significant. This demonstrates a limitation of the Monte Carlo approach to finding under-coupled metabolite pairs – it cannot locate with high confidence metabolites that are relatively scarcely connected individually.

## Conclusion

This study presents the first calculations of metabolite coupling for curated, genome-scale metabolic networks. As expected, metabolite coupling is dominated by a few highly connected pairs (H^+^/H_2_O, ATP/ADP, etc.). However, it was also shown that the coupling of metabolites with lower individual connectivity demonstrated a striking amount of regularity, with the number of reactions shared by pairs of metabolites in a given metabolic network closely approximating a line on a log-log plot. We also probed the network to discover which metabolite pairings occurred much more or less than would be expected from their individual connectivities alone. We found that the highly connected metabolites contributed a disproportionate percentage of the enriched coupling interactions in that highly connected metabolites are *more *connected to each other than would be expected from their individual connectivities alone. These preferentially coupled pairs of metabolites highlight chemical relationships between molecules. In this study, it is interesting that all of the top 15 most coupled metabolites have at least one other metabolite with which they preferentially couple in *E. coli*, highlighting the importance of metabolite coupling to the emergence of highly-connected compounds in metabolic networks.

## Methods

### Basics

We study the distribution of metabolite coupling in the chemically-detailed and highly curated metabolic networks of *E. coli *[2], *S. cerevisiae *[22], *Helicobacter pylori *[19], *Staphylococcus aureus *[20], *M. barkeri *[21], and the human cardiac mitochondrion [3]. We derived the stoichiometric matrix **S **from the publicly-available reaction lists for each organism. Each row of **S **represents a metabolite, each column represents a reaction, and each element is a stoichiometric coefficient. If a compound existed in more than one cellular compartment (for example, the lysosome), the row(s) representing a given compound in any compartment(s) other than the cytosol were added to the cytosolic row and then eliminated. This pre-processing of **S **assured that the coupling of two metabolites was considered identically regardless of the sub-cellular localization of the metabolites.

### Calculating the metabolite coupling matrix

The binary form of **S**, termed **Ŝ**, was formed for each network by replacing every non-zero element in **S **with unity. The symmetric "metabolite coupling matrix" **M **was then computed for each network by Eq. 1.

**M**=**ŜŜ**^T ^    (1)

The density of each **M **is defined as the number of non-zero elements divided by the total number of elements in the matrix. In order to visualize the entire range of data with meaningful size and greyscale differences differences, particularly between zero and small numbers, each element of **M **was transformed for figure 1 such that new value = log_2_(old value + 1)/(number of unique metabolite pairs in network).

Figure 2 uses the binary form of **M**, created by replacing all non-zero elements with 1, and was constructed in Matlab (The Mathworks, Inc.) using the imagesc function. All numerical calculations were done with the same software.

### Metabolite coupling and connectivity (Figures 3, 4, 5, 6)

For each network, the number of metabolite pairs (y-axis) that participate together in a given number of reactions (x-axis) was plotted against that number of reactions. The leftmost 10 points were used to calculate a best-fit line on a log-log scale for the less connected metabolites (those that participate in 1 to 10 reactions together). The same procedure was done for individual metabolites, except that points 2 to 11 were used to compute the best-fit line because relatively few metabolites participate in only one reaction. The best-fit lines were calculated in Excel (Microsoft).

The number of unique coupling interactions for each metabolite was computed by first sorting the rows and columns of each **M **separately, according to the ordering of the diagonal elements, such that the most connected metabolites occupy the first rows and columns. The number of non-zero elements in each row of the sorted **M **was plotted against the row number in a cumulative fashion and normalized by dividing by the total number of non-zero elements in the matrix.

### Preferentially coupled/uncoupled metabolites

Monte Carlo sampling was used to find the distribution of possible off-diagonal elements of **M**, given the diagonal elements. This procedure elucidates the range of reactions in which two metabolites couple given the number of reactions in which they occur individually. For each off-diagonal element m_ij_, we randomly computed a value based on the diagonal elements m_ii _and m_jj_. We constructed two vectors of m_ii _and m_jj _random unique integers between 1 and the total number of reactions in the network. These vectors are taken to represent the random reactions in which each metabolite individually participates. The overlap between them gives the randomized value for m_ij_. For example, take a hypothetical network with a total of 10 reactions, m_11 _= 4, and m_22 _= 3. Then, we pick 2 sets of random, unique integers between 1 and 10, one of 4 integers and one of 3: [1 4 2 8]. [2 3 1]. The integers 1 and 2 appear in both of these vectors, so the random value for m_12 _is 2. When this process is repeated many times (we used 10,000 randomizations for each off-diagonal element of **M **for each network), the random value converges to (m_ii_)(m_jj_)/(number of reactions). Importantly, actually performing the randomizations produces a distribution of all possible values for each m_ij_. By sorting the distribution of 10,000 random coupling values and noting how many random values are greater (less) than m_ij_, we estimate the probability of all pairs of two metabolites coupling as often (rarely) as they do by random chance.

## Authors' contributions

SAB carried out the computations described in the manuscript, analyzed the results, and drafted the manuscript. NDP helped perform computations, analyze the results and draft the manuscript. BOP conceived of the study, helped analyze the results, and helped draft the manuscript. All authors read and approved the final manuscript.

## Figures and Tables

**Table 1:** Basic metabolic network statistics.

	*E. coli*	*S. cerevisiae*	*H. pylori*	*S. aureus*	Mitochondrion	*M. barkeri*
Number of compounds	621	642	404	554	145	544
Number of reactions	802	861	417	588	130	564
Density of **M**	2.2%	2.2%	3.1%	2.4%	7.8%	2.3%

**Table 2:** Coupling of prominent metabolites. The number of metabolites that couple with each compound. In the *E. coli *metabolic network, the proton participates in at least one reaction with 479 other unique metabolites. The ordering of metabolites is based on the number of reactions they participate in individually, averaged across all networks. Thus, the number of unique metabolites that couple with a given metabolite do not strictly have to decrease down a column, although it usually does.

	Number of unique metabolites with which each prominent metabolite couples					
Metabolite	*E. coli*	*S. cerevisiae*	*H. pylori*	*S. aureus*	mitochondrion	*M. barkeri*

H^+^	479	490	291	400	97	402
H_2_O	368	361	214	275	65	265
ATP	220	262	135	214	59	215
ADP	179	161	114	142	42	134
P_i_	184	161	123	132	34	165
PP_i_	140	175	101	143	28	166
CO_2_	116	125	90	122	33	104
NAD^+^	126	121	44	108	34	86
NADP^+^	92	144	71	97	14	86
NADH	123	116	37	111	34	78
CoA	72	109	46	95	49	59
NADPH	90	143	68	105	14	83
AMP	72	126	39	80	24	108
glu	84	80	57	71	17	91
NH_4_	80	88	58	68	14	64

**Table 3:** Preferentially coupled metabolite pairs in *E. coli*. The preferentially coupled metabolite pairs in the *E. coli *metabolic network, computed as described in the text. All of these pairs occur more often in the real network then in any of 10,000 randomizations, for an effective p value of 0.

H+	ATP
H+	ADP
H+	Nicotinamide adenine dinucleotide
H+	Nicotinamide adenine dinucleotide – reduced
H+	CO2
H+	Nicotinamide adenine dinucleotide phosphate
H+	Nicotinamide adenine dinucleotide phosphate – reduced
H2O	Phosphate
H2O	Ammonium
ATP	Phosphate
ATP	ADP
ATP	Diphosphate
ATP	AMP
Phosphate	ADP
Nicotinamide adenine dinucleotide	Nicotinamide adenine dinucleotide – reduced
Diphosphate	AMP
Diphosphate	5-Phospho-alpha-D-ribose 1-diphosphate
Diphosphate	Farnesyl diphosphate
CO2	Malonyl-[acyl-carrier protein]
CO2	Acetoacetyl-ACP
Nicotinamide adenine dinucleotide phosphate	Nicotinamide adenine dinucleotide phosphate – reduced
Nicotinamide adenine dinucleotide phosphate	Malonyl-[acyl-carrier protein]
Nicotinamide adenine dinucleotide phosphate	Acetoacetyl-ACP
Pyruvate	Phosphoenolpyruvate
Nicotinamide adenine dinucleotide phosphate – reduced	Malonyl-[acyl-carrier protein]
Nicotinamide adenine dinucleotide phosphate – reduced	Acetoacetyl-ACP
L-Glutamate	2-Oxoglutarate
L-Glutamate	L-Glutamine
Coenzyme A	Acetyl-CoA
Coenzyme A	Succinyl-CoA
acyl carrier protein	Malonyl-[acyl-carrier protein]
acyl carrier protein	Acetoacetyl-ACP
acyl carrier protein	Myristoyl-ACP (n-C14:0ACP)
acyl carrier protein	Hexadecenoyl-ACP (n-C16:1ACP)
acyl carrier protein	R-3-hydroxy-myristoyl-ACP
acyl carrier protein	Tetradecenoyl-ACP (n-C14:1ACP)
acyl carrier protein	Octadecenoyl-ACP (n-C18:1ACP)
acyl carrier protein	Dodecanoyl-ACP (n-C12:0ACP)
acyl carrier protein	Palmitoyl-ACP (n-C16:0ACP)
O2	Hydrogen peroxide
D-Glucose	Maltohexaose
D-Glucose	Maltopentaose
D-Glucose	Maltose
D-Glucose	Maltotetraose
D-Glucose	Maltotriose
Ubiquinol-8	Ubiquinone-8
Menaquinone 8	Menaquinol 8
Succinate	Fumarate
L-Aspartate	L-Asparagine
CMP	CDPdiacylglycerol (E coli)
CMP	CMP-3-deoxy-D-manno-octulosonate
2-Demethylmenaquinone 8	2-Demethylmenaquinol 8
GTP	GDP
Malonyl-[acyl-carrier protein]	Acetoacetyl-ACP
Oxaloacetate	L-Malate
Reduced thioredoxin	Oxidized thioredoxin
Glyoxylate	Glycolate
Flavin adenine dinucleotide oxidized	Flavin adenine dinucleotide reduced
S-Adenosyl-L-methionine	S-Adenosyl-L-homocysteine
tetradecanoate (n-C14:0)	Hexadecanoate (n-C16:0)
tetradecanoate (n-C14:0)	tetradecenoate (n-C14:1)
tetradecanoate (n-C14:0)	hexadecenoate (n-C16:1)
tetradecanoate (n-C14:0)	octadecenoate (n-C18:1)
Hexadecanoate (n-C16:0)	tetradecenoate (n-C14:1)
Hexadecanoate (n-C16:0)	hexadecenoate (n-C16:1)
Hexadecanoate (n-C16:0)	octadecenoate (n-C18:1)
5,6,7,8-Tetrahydrofolate	5,10-Methylenetetrahydrofolate
5,6,7,8-Tetrahydrofolate	10-Formyltetrahydrofolate
tetradecenoate (n-C14:1)	hexadecenoate (n-C16:1)
tetradecenoate (n-C14:1)	octadecenoate (n-C18:1)
hexadecenoate (n-C16:1)	octadecenoate (n-C18:1)
hexadecenoate (n-C16:1)	acyl-glycerophosphoglycerol (E.coli)
octadecenoate (n-C18:1)	acyl-glycerophosphocholine
UDPglucose	UDPgalactose
Maltohexaose	Maltopentaose
Maltohexaose	Maltoheptaose
Phosphatidylglycerol (Ecoli)	acyl phosphatidylglycerol (E.coli)
Maltopentaose	Maltotetraose
Pyridoxal 5'-phosphate	Pyridoxamine 5'-phosphate
Pyridoxal 5'-phosphate	Pyridoxal
NMN	Nicotinamide
Reduced glutathione	Glutathionylspermidine
Reduced glutathione	(R)-S-Lactoylglutathione
Isopentenyl diphosphate	Farnesyl diphosphate
Isopentenyl diphosphate	Dimethylallyl diphosphate
Isopentenyl diphosphate	Geranyl diphosphate
D-Gluconate	2-Dehydro-D-gluconate
Spermidine	Glutathionylspermidine
3-Methyl-2-oxobutanoate	L-Valine
CMP-3-deoxy-D-manno-octulosonate	KDO-lipid IV(A)
Trimethylamine N-oxide	trimethylamine
Dimethyl sulfoxide	Dimethyl sulfide
alpha,alpha'-Trehalose 6-phosphate	Trehalose
1-Pyrroline-5-carboxylate	L-Proline
UDP-2,3-bis(3-hydroxytetradecanoyl)glucosamine	2,3-Bis(3-hydroxytetradecanoyl)-beta-D-glucosaminyl 1-phosphate
acyl-glycerophosphoethanolamine (E.coli)	sn-Glycero-3-phosphoethanolamine
acyl-glycerophosphoglycerol (E.coli)	Glycerophosphoglycerol
Carnitinyl-CoA	crotonobetainyl-CoA
Carnitinyl-CoA	L-Carnitine
N-Acetyl-D-mannosamine 6-phosphate	N-Acetyl-D-mannosamine
2-keto-4-methylthiobutyrate	2,3-diketo-5-methylthio-1-phosphopentane
Biotin	d-biotin d-sulfoxide
Nitrite	Nitrate
N1-(5-Phospho-D-ribosyl)glycinamide	N2-Formyl-N1-(5-phospho-D-ribosyl)glycinamide
Orotate	(S)-Dihydroorotate
4-(1-D-Ribitylamino)-5-aminouracil	6,7-Dimethyl-8-(1-D-ribityl)lumazine
Glycine betaine	Betaine aldehyde
L-Glutamyl-tRNA(Glu)	tRNA (Glu)

**Table 4:** Preferentially uncoupled metabolite pairs in *E. coli*. The top preferentially uncoupled metabolite pairs in the *E. coli *metabolic network.

**Metabolite**	**Metabolite**	**p-value**
H+	Pyruvate	0.0001
ATP	Nicotinamide adenine dinucleotide phosphate	0.0001
ATP	Nicotinamide adenine dinucleotide phosphate – reduced	0.0001
ADP	Nicotinamide adenine dinucleotide	0.0001
ADP	Diphosphate	0.0001
ADP	Nicotinamide adenine dinucleotide – reduced	0.0001
ADP	Nicotinamide adenine dinucleotide phosphate – reduced	0.0001
ATP	Nicotinamide adenine dinucleotide – reduced	0.0002
Phosphate	Nicotinamide adenine dinucleotide – reduced	0.0002
H2O	2-Oxoglutarate	0.0003
Phosphate	Nicotinamide adenine dinucleotide	0.0003
H+	Phosphoenolpyruvate	0.0004
ADP	Nicotinamide adenine dinucleotide phosphate	0.0004
ADP	Pyruvate	0.0005
H+	D-Glucose	0.0006
ATP	Pyruvate	0.0006
H+	2-Oxoglutarate	0.0007
ATP	Nicotinamide adenine dinucleotide	0.0007
Phosphate	Pyruvate	0.0009
